# Feasibility of Extrapolating Randomly Taken Plasma Samples to Trough Levels for Therapeutic Drug Monitoring Purposes of Small Molecule Kinase Inhibitors

**DOI:** 10.3390/ph14020119

**Published:** 2021-02-04

**Authors:** Ruben A. G. van Eerden, Esther Oomen-de Hoop, Aad Noordam, Ron H. J. Mathijssen, Stijn L. W. Koolen

**Affiliations:** 1 Department of Medical Oncology, Erasmus MC Cancer Institute, 3015GD Rotterdam, The Netherlands; e.oomen-dehoop@erasmusmc.nl (E.O.-d.H.); a.noordam@erasmusmc.nl (A.N.); a.mathijssen@erasmusmc.nl (R.H.J.M.); s.koolen@erasmusmc.nl (S.L.W.K.); 2 Department of Hospital Pharmacy, Erasmus MC, University Medical Center, 3015GD Rotterdam, The Netherlands

**Keywords:** small molecule kinase inhibitor, trough level, C_min_, therapeutic drug monitoring, implementation

## Abstract

Small molecule kinase inhibitors (SMKIs) are widely used in oncology. Therapeutic drug monitoring (TDM) for SMKIs could reduce underexposure or overexposure. However, logistical issues such as timing of blood withdrawals hamper its implementation into clinical practice. Extrapolating a random concentration to a trough concentration using the elimination half-life could be a simple and easy way to overcome this problem. In our study plasma concentrations observed during 24 h blood sampling were used for extrapolation to trough levels. The objective was to demonstrate that extrapolation of randomly taken blood samples will lead to equivalent estimated trough samples compared to measured C_min_ values. In total 2241 blood samples were analyzed. The estimated C_trough_ levels of afatinib and sunitinib fulfilled the equivalence criteria if the samples were drawn after T_max_. The calculated C_trough_ levels of erlotinib, imatinib and sorafenib met the equivalence criteria if they were taken, respectively, 12 h, 3 h and 10 h after drug intake. For regorafenib extrapolation was not feasible. In conclusion, extrapolation of randomly taken drug concentrations to a trough concentration using the mean elimination half-life is feasible for multiple SMKIs. Therefore, this simple method could positively contribute to the implementation of TDM in oncology.

## 1. Introduction

During the last decades many small molecule kinase inhibitors (SMKIs) have been developed and become widely available for the treatment of multiple types of malignancies. SMKIs changed the treatment paradigm towards a targeted therapy approach with oral anticancer drugs instead of the traditional intravenous chemotherapy as cancer treatment. For many SMKIs an exposure–response relation has been published in recent literature [1,2,3]. Despite the narrow therapeutic window and large inter-patient variability, they are still prescribed at a fixed dose [4,5,6,7,8,9,10]. Consequently, the risk for severe toxicity due to high exposure or ineffectiveness due to low exposure, is high.

Personalized dosing by therapeutic drug monitoring (TDM) is a way to address and possibly solve the problem of under- or overdosing patients [5]. TDM based dosing is already standard clinical practice for many antibiotic and immunosuppressive drugs and is increasingly being explored for oncological drugs [1,2,11,12,13]. It has been proven to be feasible in oncology and could improve the treatment outcome [4,6,14]. However, logistical issues of TDM often hamper the introduction of personalized dosing.

Targets used for TDM are based on plasma exposure that is measured as AUC (area under the concentration-time curve) or as trough concentration (C_trough_) [2,5,15]. The trough concentration is usually the concentration measured immediately before the administration of a new dose. This specific time point as the target for TDM causes some logistical difficulties. Outpatient visits are planned randomly during the day and a part of the patients take their drugs during the evening when it is very difficult to withdraw blood. A real C_trough_ sample is therefore almost impossible to measure. This difficulty could potentially be overcome by the estimation of the C_trough_ from a randomly taken sample over time.

A simple method for this estimation might be extrapolation of randomly measured concentration to a trough concentration using the mean population terminal half-life of a drug. This does not require specific patient characteristics. This method was thoroughly investigated by Wang et al. for imatinib and was found feasible [16]. This method is easy to implement in the daily routine. The objective of our study was to investigate whether this method is also feasible for more SMKIs, as this might be helpful for the implementation of TDM in daily oncological practice. 

## 2. Results

In total 2105 blood samples with corresponding trough samples were analyzed. Samples were taken from patients participating in nine different prospective pharmacokinetic studies (Table 1) [17,18,19,20,21,22,23,24,25]. Table 2 presents per drug pharmacokinetic parameters used, analyzed cohorts, relative differences and 90% confidence interval (90%CI) of the relative difference. 

### 2.1. Afatinib

**Figure 1:**
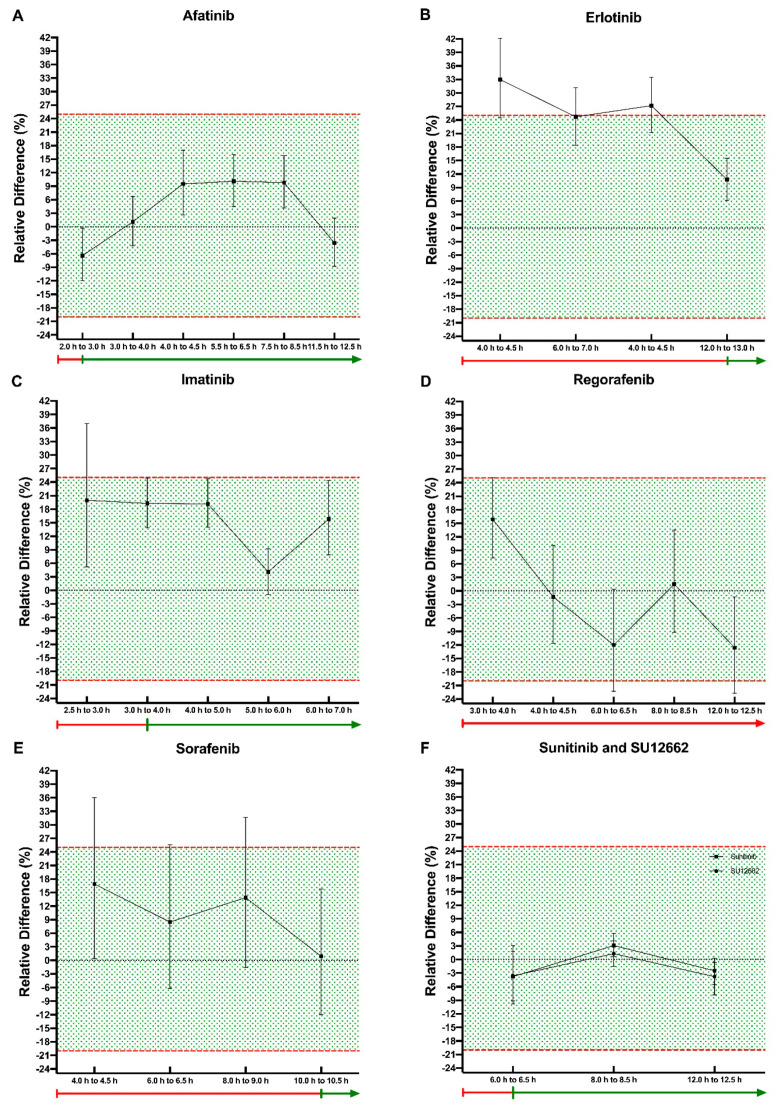
Observed relative differences with corresponding 90% confidence interval of the estimated trough concentration compared to measured trough concentration per drug over time: (**A**) Relative differences observed in the afatinib cohort; (**B**) Relative differences observed in the erlotinib cohort; (**C**) Relative differences observed in the imatinib cohort; (**D**) Relative differences observed in the regorafenib cohort; (**E**) Relative differences observed in the sorafenib cohort; (**F**) Relative differences observed in the sunitinib and SU12662 cohorts). The green fields represent the values of bio-equivalence. The line below the *x*-axis represents our recommended extrapolation intervals between previous drug intake and blood withdrawal for extrapolation (i.e., red: not recommended, green: extrapolation useful).

In total, 271 samples were included from 13 patients drawn between 2 h and 12.5 h after intake of afatinib [25]. Based on time after intake and available pharmacokinetic (PK) samples obtained during the prospective PK study, samples were divided into six cohorts based on the available measurements: 2 h to 3 h, 3 h to 4 h, 4 h to 4.5 h, 5.5 h to 6.5 h, 7.5 h to 8.5 h and 11.5 h to 12.5 h, respectively. Relative differences and corresponding 90% confidence interval remained within the range of −20% to +25% for all cohorts (Figure 1A). The relative difference and 90%CI per cohort are presented in Table 2. 

### 2.2. Erlotinib

For erlotinib 455 samples from 59 patients were available for analysis [23,24]. The samples were sorted according to time after once daily intake of the erlotinib and available PK samples obtained during the prospective PK study. The 12 h to 13 h cohort was the only cohort in which the relative differences remained within the −20% to +25% range (RD: 10.8%; 90%CI: 6.2% to 15.5%). The other cohorts consisted of samples taken between 4 h to 4.5 h, 6 h to 7 h and 8 h to 8.5 h (Figure 1B). Detailed results are displayed in Table 2.

### 2.3. Imatinib

In total, 617 imatinib samples were used in the analysis [18,19]. The relative difference for the first cohort of samples taken at 2.5 h to 3 h after intake of once daily imatinib was 20.0% (90%CI: 5.2% to 37.0%). The relative differences and corresponding 90% confidence interval of the other cohorts remained within the range of −20% to +25% (cohort 3 h to 4 h RD: 19.3% (90%CI: 13.9% to 24.9%); cohort 4 h to 5 h RD: 19.2% (90%CI: 14.0% to 24.7%); cohort 5 h to 6 h RD: 4.1% (90%CI: −0.9% to 9.2%); cohort 6 h to 7 h RD: 15.8% (90%CI: 7.9% to 24.3%)) (Figure 1C, Table 2).

### 2.4. Regorafenib

For regorafenib, 309 samples from 22 patients were available for analysis [17]. The samples were divided into the following five cohorts based on available PK samples obtained during the prospective PK study: 3 h to 4 h, 4 h to 4.5 h, 6 h to 6.5 h, 8 h to 8.5 h and 12 h to 12.5 h. The relative difference and corresponding 90% confidence interval of the 4 h to 4.5 h cohort and 8 h to 8.5 h cohort remained within the range of −20% to +25% RD (RD: −1.3%; 90%CI: −11.6% to 10.1% and RD: 1.5%; 90%CI: −9.2% to 13.5%, respectively). None of the other cohorts met the predefined equivalence requirements (Figure 1D, Table 2).

### 2.5. Sorafenib

In total, 128 samples were included from 16 patients drawn between 4 h and 10.5 h after intake of twice daily sorafenib [20]. Only the cohort of samples drawn between 10 h and 10.5 h met the predefined definition of equivalence (RD 0.9% (90%CI: −12.0% to 15.8%)). None of the other cohorts met the equivalence requirements with an RD varying between 8.5% and 13.9% with an upper limit of the 90%CI above the 25% (Figure 1E, Table 2).

### 2.6. Sunitinib and SU12662

For sunitinib and the active metabolite SU12662, 231 samples were taken between 6 h and 13 h after intake of sunitinib [21,22]. Samples were divided into three cohorts based on available PK samples obtained during the prospective PK study (Table 2). The relative differences of sunitinib in the analyzed cohorts varied between −3.8% and 3.1% while the relative difference of SU12662 varied between −3.6% and 3.8%. The corresponding 90% confidence interval of all cohorts remained within the range of −20% to +25% (Figure 1F). 

## 3. Discussion

In this study we have shown that for most analyzed drugs extrapolation of a randomly taken plasma sample to a trough concentration is feasible. Nevertheless, each drug has its own time point after which the extrapolated C_trough_ was equivalent to the truly measured C_trough_. Based on our results we formulated for each investigated drug a recommended interval between previous drug intake and blood withdrawal, which are summarized in Table 3 and Figure 1. For afatinib this interval is recommended to be at least 2 h and our recommended intervals are 3 h, 6 h and 12 h for imatinib, sunitinib and erlotinib, respectively. Extrapolation of sorafenib samples is feasible 10 h after intake of the last dose. However, this is thus only of limited benefit since sorafenib has a dose interval of 12 h. Extrapolation of regorafenib samples seems feasible for samples taken between 3–4.5 h and 8–8.5 h after the last dose. However, given the inconsistency in fulfillment of the predefined equivalence requirements we do not recommend extrapolation of randomly taken regorafenib samples to trough samples. If regorafenib samples are extrapolated, results should be interpreted with caution. 

After which time point extrapolation of a blood sample is feasible differs depending on the pharmacokinetic characteristics of each individual drug. Most of the analyzed SMKIs (e.g., sunitinib, imatinib, erlotinib, afatinib) are characterized by a first-order elimination [26,27,28,29]. The equation used in our analyses is based on the pharmacokinetic equation that describes first-order elimination, and is therefore likely to be able to estimate a C_trough_ from a randomly taken sample. Although the equation should theoretically approximate the C_trough_, we still observed differences between the estimated C_trough_ and the measured C_trough_. This might be explained by the deviation of the standardized K_e_ used in our analyses compared with the K_e_ of each individual in clinical practice. We calculated the K_e_ based on the mean elimination half-life described in the summary of product characteristics (SMPC) that does not take into account patient factors that could influence this PK parameter. Therefore, clinicians should be cautious with the use of this equation and population mean values for the terminal half-life when a drug–drug interaction (e.g., a strong induction of inhibition of CYP3A4) or genetic polymorphism (e.g., CYP3A4*22) affects the drug metabolism [10,30]. Another way of estimating the K_e_ is used by Wang et al. [16], who calculated K_e_ based on a population PK model of imatinib. However, the deviation between the estimated non corrected C_trough_ and the measured C_trough_ for the highest sampling interval (i.e., 17 h) described by Wang et al. [16] was 33.2% for the typical K_e_ while the deviation in all our cohorts ranged between 4% and 20% which is remarkably smaller. Therefore, it could be suggested that our way of K_e_ calculation leads to a more accurate C_trough_ estimation. However, caution is warranted since the elimination half-life in the SMPC is an average half-life that might be accurate in a large average population but might strongly differ from the elimination half-life of a certain other specific population (e.g., other ethnicities than the majority of Caucasian patients in our studies) [31]. Using an ethnicity specific K_e_ might therefore also be an option and might be more accurate. 

Nonetheless, it could be speculated that K_e_ estimation based on elimination half-life derived from the SMPC leads to a more standardized way of extrapolation since the SMPC is freely accessible for all clinicians while K_e_ calculations based on the previously described data (e.g., of a specific population) might vary depending on the used reference. 

Extrapolation of randomly taken samples from patients treated with regorafenib and sorafenib is less accurate in estimating the C_trough_. This is probably caused by the enterohepatic recycling of regorafenib and sorafenib [17,32,33]. Enterohepatic recycling could cause a second peak in the plasma concentration-time curve [34]. A second peak in this curve was not taken into account in our analyses which causes a less accurate estimation of the C_trough_.

To improve the estimation of the C_trough_ concentration, it could also be suggested to add a correction factor to the algorithm for each drug. Wang et al. [16] applied a correction factor ranging between 0.752 and 0.885 for samples drawn before the following intake of imatinib that reduced the deviation of the estimated C_trough_ from the measured C_trough_. A correction factor might also be applicable for our imatinib data given the, in most cohorts observed, relative differences of around the 20% between the estimated C_trough_ and the true C_trough_.

As this method of extrapolation is feasible for most of the investigated drugs, this method could also be used for TDM purposes in oncological care. TDM is used to personalize the treatment by adjusting the dose based on the plasma drug concentrations of the patient [5]. By personalizing the dose, TDM aims to reduce underexposure and overexposure to a drug which improves the treatment outcome [5]. Given the random timing of outpatient visits and blood withdrawals, targets used for TDM, as AUC or trough concentrations, are unfortunately difficult to determine in clinical practice. Since feasibility of this extrapolation method has been proven, blood withdrawals can from now on be performed during a bigger window of time. This could resolve the aforementioned logical issues related to TDM in the daily care of cancer patients. However, when using this extrapolation method for TDM purposes a pitfall might be misinterpretation of the drug exposure based on the estimated C_trough_. Because of the relative differences observed between the measured and estimated C_trough_, a patients C_trough_ might be overestimated or underestimated. Misinterpretation could lead to an inaccurate TDM advice. Overestimation could lead to falsely maintaining the currently used dose that could lead to less effectiveness while underestimation might lead to a redundant dose increment that could lead to increased toxicity and costs. However, this pitfall might be solved by repeated TDM sampling as proposed in the therapeutic drug monitoring protocol of the Dutch Pharmacology Oncology Group [11].

Bayesian estimation based on a known PK-model is another method which could be used for the estimation of a C_trough_ level. Bayesian estimates permit flexible blood withdrawals and provide the opportunity to take patient characteristics into account [15,35]. These kind of adjustments are not possible with the formula used for our analysis [36]. Nevertheless, Bayesian estimates also have their drawbacks. To use this method prior awareness of patients’ characteristics, which are sometimes not available or hard to determine, is desirable, because otherwise the estimation will be guided towards the typical population mean [3,15]. Eventually this will lead to loss of individual information of the patient. Moreover, a previously described PK model, specialized software and knowledge is needed to execute and interpret these calculations [3,36]. In contrast to Bayesian estimates, the method we used is simple to execute in clinical practice and does not depend on which model has been chosen.

In conclusion, our analysis proves that a simple equation using the mean elimination half-life can accurately estimate the C_trough_ from a randomly taken plasma sample for SMKIs which are not pharmacokinetically characterized by enterohepatic recycling. This method could reduce the variability and standardize the method used for C_trough_ estimation as well as simplify the implementation of TDM in the daily practice of oncology. Further studies are warranted to evaluate the feasibility of the analyzed method when used for other SMKIs. 

## 4. Materials and Methods

Prospective pharmacokinetic trials performed between 2000 and 2021 at the Erasmus MC Cancer Institute, Rotterdam, The Netherlands, investigating SMKIs were used in our analysis. 

Studies were included for analysis if they investigated the pharmacokinetics (PK) of SMKIs, at least two blood samples within a 24 h time frame were taken including the trough concentration and patients were treated with the approved dose. Moreover, the exact time of drug intake and blood withdrawal had to be known. Studies or samples were excluded if an intervention that influenced the metabolism of the drug was performed during the blood sampling (e.g., CYP3A4 induction by concomitant study drugs). Blood samples taken before T_max_ as stated in the SMPC were also excluded from analysis. If a time range for T_max_ was stated in the SMPC the lower boundary was used as cut-off point.

The cohort of patients used for this analysis was obtained from nine prospective studies (Table 1). All studies were approved by the medical ethics committee of the Erasmus MC (MEC2004-018, MEC2008-256, MEC2009-302, MEC2012-138, MEC2014-046, MEC2016-165, MEC2016-590, MEC2017-251, MEC2017-490) and all patients gave written informed consent.

The feasibility of extrapolating randomly taken blood samples will be assessed using the criteria of equivalence (i.e., 90%CI of the geometric mean ratio between 0.8 and 1.25) compared to the measured C_min_ value.

### 4.1. Pharmacokinetic Equation

The PK profile of most of the SMKIs is characterized by first-order elimination [26,27,28,29]. Therefore, the concentration at a random time point during the elimination phase (T2) can be estimated based on the concentration measured at another time point during the elimination phase (T1) using the following pharmacokinetic equation:

$$C_{t2}=C_{t1}\;exp\left(-ke\times \Delta t\right)$$

And this equation can be rewritten to:

Ct2=Ct1 exp(−0.693t12×Δt)

In this equation *C_t_*_1_ represents the plasma drug concentration measured at the moment of blood withdrawal (i.e., a random time-point). T^1^/_2_ represents the elimination half-life and is taken from the SMPC for each drug. The factor ∆*t* represents the time between the moment of blood withdrawal (i.e., known and measured drug concentration) and the moment of the next drug intake (time point of C_trough_). By filling in the aforementioned factors the product *C_t_*_2_ is calculated which represents the estimated trough concentration.

### 4.2. Statistical Analysis

IBM SPSS statistics (IBM Corporation, version 25.0. Armonk, NY, USA) was used to build the database and to perform the statistical analyses. The estimated C_trough_ (C_trough estimated_) was calculated based on the aforementioned equation per included drug measurement. Log transformation was performed on each C_trough_ and C_trough estimated_, because a lognormal distribution was assumed for the plasma concentrations. Samples were grouped according to drug and relative time of sample collection. Per drug and per time-point a paired T-test was performed between the C_trough_ and C_trough estimated_ on the log transformed data. Mean differences and 90% confidence intervals of these differences were calculated. Exponentiation of the mean differences and 90%CI provided the geometric mean ratio and corresponding 90%CI which can be interpreted as the relative differences in percentages. Equivalence between the C_trough_ and C_trough estimated_ was shown if the 90%CI was within the lower boundary of –20% and upper boundary of +25% (i.e., geometric mean ratio CI within 0.80 and 1.25). These boundaries are based on the EMA guideline of bio-equivalence studies [37]. Based on this guideline a deviation of the true measured C_trough_ which is outside this window could lead to a clinically relevant deviation in exposure. Boundaries smaller than −20% and +25% are difficult given the allowed deviation in measurement of bioanalytical methods used [38]. Extrapolating TDM samples to estimate the trough concentration was considered feasible for clinical implementation if the equivalence requirements were met.

## Figures and Tables

**Table 1:** Used prospective pharmacokinetic trials.

Title of Prospective Pharmacokinetic Study	Reference
A long-term prospective population pharmacokinetic study on imatinib plasma concentrations in GIST patients	[19]
Environmental and genetic factors affecting transport of imatinib by OATP1A2	[18]
Predictive Value of CYP3A and ABCB1 Phenotyping Probes for the Pharmacokinetics of Sunitinib: the ClearSun Study	[22]
Relationship Between Sunitinib Pharmacokinetics and Administration Time: Preclinical and Clinical Evidence	[21]
Influence of the Acidic Beverage Cola on the Absorption of Erlotinib in Patients With Non-Small-Cell Lung Cancer	[23]
Influence of Cow’s Milk and Esomeprazole on the Absorption of Erlotinib: A Randomized, Crossover Pharmacokinetic Study in Lung Cancer Patients	[24]
Influence of Probenecid on the Pharmacokinetics and Pharmacodynamics of Sorafenib	[20]
Influence of the Proton Pump Inhibitor Esomeprazole on the Bioavailability of Regorafenib: A Randomized Crossover Pharmacokinetic Study	[17]
The effects of esomeprazole on the bioavailability of afatinib in patients with non-small-cell lung cancer	[25]

**Table 2:** Investigated cohorts and relative differences of the estimated trough concentration compared to measured trough concentration.

SMKI	T_max_ (h)	T_1/2_ (h)	DI (h)	Patients (*n*)	Time after Intake (h)	Blood Withdrawal (n)	RD (%)	90%CI RD (%)
Afatinib	2 to 5	37	24	13	2.00 to 2.98	66	−6.4	−12.0 to −0.3
				13	3.00 to 3.98	70	1.1	−4.2 to 6.7
				13	4.00 to 4.13	30	9.5	2.6 to 17.0
				13	5.50 to 6.48	36	10.1	4.5 to 16.0
				13	7.50 to 8.30	35	9.8	4.2 to 15.8
				13	11.50 to 12.73	34	−3.6	−8.8 to 1.9
Erlotinib	4	36	24	55	4.00 to 4.27	98	33.0	24.5 to 42.1
				59	5.90 to 6.63	120	24.7	18.4 to 31.2
				59	7.87 to 8.33	119	27.2	21.2 to 33.5
				59	11.92 to 13.00	118	10.8	6.2 to 15.5
Imatinib	2.5	18	24	25	2.58 to 2.98	37	20.0	5.2 to 37.0
				61	3.00 to 3.98	200	19.3	13.9 to 24.9
				61	4.00 to 4.92	176	19.2	14.0 to 24.7
				58	5.00 to 6.00	150	4.1	−0.9 to 9.2
				34	6.02 to 7.08	54	15.8	7.9 to 24.3
Regorafenib	3 to 4	20 to 30	24	22	3.00 to 3.98	106	15.9	7.3 to 25.1
				22	4.00 to 4.58	47	−1.3	−11.6 to 10.1
				22	5.90 to 6.50	52	−12.0	−22.3 to 0.4
				22	7.88 to 8.50	52	1.5	−9.2 to 13.5
				22	11.82 to 12.10	52	−12.6	−22.7 to −1.3
Sorafenib	3	25 to 48	12	16	3.98 to 4.22	32	16.9	0.4 to 36.0
				16	5.97 to 6.15	32	8.5	−6.2 to 25.6
				16	7.93 to 8.43	32	13.9	−1.6 to 31.7
				16	9.93 to 10.25	32	0.9	−12.0 to 15.8
Sunitinib	6 to 12	40 to 60	24	17	6.00 to 6.27	30	−3.8	−9.1 to 1.8
				75	7.63 to 8.67	149	3.1	0.6 to 5.8
				24	11.95 to 12.42	52	−2.5	−5.6 to −0.7
SU12662	6 to 12	80 to 110	24	17	6.00 to 6.27	30	−3.6	−9.8 to 3.1
				75	7.63 to 8.67	148	1.3	−1.5 to 4.1
				23	11.95 to 12.42	52	3.8	−7.8 to 0.3

Abbrevations: SMKI = Small Molecule Kinase Inhibitor; T_max_ = time point when maximum concentration is reached in plasma; T_1/2_ = elimination half-life of drug; DI = dosing interval; RD = Relative Difference; CI = confidence interval; SU12662 = active metabolite of sunitinib.

**Table 3:** Recommended intervals between previous drug intake and blood withdrawal.

Drug	Recommended Interval
Afatinib	≥2 h
Erlotinib	≥12 h
Imatinib	≥3 h
Regorafenib	Not recommended
Sorafenib	≥10 h
Sunitinib (and SU12662)	≥6 h

Abbrevations: SU12662 = active metabolite of sunitinib.

## Data Availability

The data that support the findings of this study are available from the corresponding author (RvE) upon reasonable request.
